# Genome-wide matching of genes to cellular roles using guilt-by-association models derived from single sample analysis

**DOI:** 10.1186/1756-0500-5-370

**Published:** 2012-07-23

**Authors:** Jeff A Klomp, Kyle A Furge

**Affiliations:** 1 Center for Cancer Genomics and Computational Biology, Van Andel Research Institute, Grand Rapids, MI, USA

**Keywords:** Gene set enrichment analysis, Gene module, Co-regulated gene sets, Gene prediction, Protein function, Olfactory receptors, Mitochondria, Lysosome, Oxidative phosphorylation

## Abstract

**Background:**

High-throughput methods that ascribe a cellular or physiological function for each gene product are useful to understand the roles of genes that have not been extensively characterized by molecular or genetic approaches. One method to infer gene function is "guilt-by-association", in which the expression pattern of a poorly characterized gene is shown to co-vary with the expression of better-characterized genes. The function of the poorly characterized gene is inferred from the known function(s) of the well-described genes. For example, genes co-expressed with transcripts that vary during the cell cycle, development, environmental stresses, and with oncogenesis have been implicated in those processes.

**Findings:**

While examining the expression characteristics of several poorly characterized genes, we noted that we could associate each of the genes with a cellular phenotype by correlating individual gene expression changes with gene set enrichment scores from individual samples. We evaluated the effectiveness of this approach using a modest sized gene expression data set (expO) and a compendium of gene expression phenotypes (MSigDBv3.0). We found the transcripts that correlated best with enrichment in mitochondrial and lysosomal gene sets were mostly related to those processes (89/100 and 44/50, respectively). The reciprocal evaluation, ranking gene sets according to correlation of enrichment with an individual gene’s expression, also reflected known associations for prominent genes in the biomedical literature (16/19). In evaluating the model, we also found that 4% of the genome encodes proteins that are associated with small molecule and small peptide signal transduction gene sets, implicating a large number of genes in both internal and external environmental sensing.

**Conclusions:**

Our results show that this approach is useful to infer functions of disparate sets of genes. This method mirrors the biological experimental approaches used by others to associate individual genes with defined gene expression changes. Moreover, the approach can be used beyond discovering genes related to a cellular process to discover meaningful expression phenotypes from a compendium that are associated with a given gene. The effectiveness, versatility, and breadth of this approach make possible its application in a variety of contexts and with a variety of downstream analyses.

## Findings

### Background

Groups of genes that are co-expressed in a subset of samples, sometimes termed gene modules, can reflect an underlying biological activity. This type of "guilt-by-association" can be a powerful method to infer a gene's cellular role. Sets of genes that are co-expressed in varying cellular states, such as transcripts that are co-expressed during different phases of the cell cycle, transcripts that vary upon certain environmental exposures, transcripts that vary during cellular or tissue differentiation, and transcripts that show conserved coregulation across different organisms can be especially insightful [1-7].

Many of the current models that describe gene co-regulation are probabilistic and can be grouped into one of two general categories: 1) models that associate gene expression changes with response to a defined experimental or biological condition (such as those described above) and 2) models that group genes by similarities in expression patterns across a compendia of gene expression profiles, often as a genomic-approach. An example of a model that falls into the first category is the work described by Hughes *et al.*, in which expression profiles from yeast that contained targeted deletion of genes with unknown functions were compared to expression profiles from yeast with targeted deletion of genes with known functions [5]. The functions of these uncharacterized genes were subsequently inferred based on similarity to the transcriptional responses of inactivation of genes with known functions. In the second category, gene pairs that are co-expressed across a series of expression profiles are used to infer similarities in biological functions. Gene networks are often constructed to relate genes to one another in a pair-wise fashion. However, the inferred functions of transcriptionally related genes are not discovered based on similarity to a defined biological perturbation as described in the first category of models [8,9]. Together, these guilt-by-association approaches have been useful to identify functions of transcripts ranging from disease-associated genes to microRNA function [10,11]. In addition, a number of other context-specific co-regulation associations have been proposed [12-17] and several guilt-by-association models have been extended to incorporate a wider variety of genomic information with the gene pair associations, such as location data [18], motif profiles [19], phenotypic data [20] and GWAS signals [21].

Gene pair associations, networks, and gene modules fit well with our understanding of the regulation of biological systems [22]. Although gene modules constructed from tumor gene expression data have proven to capture important characteristics of tumors [14,23,24], interpretation of these modules can be difficult if the genes making up the modules are not clearly linked to a cellular phenotype. Hence, a disadvantage of guilt-by-association approaches is that once a gene module is defined, the biological significance of the module is not immediately clear. While expert examination and literature curation of the genes in the module can provide insight into the biological role of the module, this is inefficient if a large number of modules are under examination or if a module contains a high proportion of genes with unknown roles. Consequently, gene set enrichment analysis approaches are used to allow researchers to understand the gene expression variability observed in their analyses in terms of pre-defined lists of genes with known biological or experimental underpinnings, termed gene sets [25,26]. Gene sets associated with a given biological process are useful tools to investigate coordinated changes in cellular processes. Large collections of gene sets have been made publicly available (*e.g*., MSigDB, GO, KEGG) and can be used with gene set enrichment approaches to find which gene sets best capture the difference in transcriptional characteristics between various environmental exposures, disease states, developmental states, or other experimental comparisons. It is therefore possible to identify the biological process associated with a transcriptional profile by comparison to well-defined gene sets.

To make explicit associations between genes and the biological processes captured by gene sets, we examined the correlations between individual fold-change gene expression values and gene set enrichment statistics in human tumor tissue samples. We explored the effectiveness of this guilt-by-association approach by genome-wide interrogation of individual genes for strongly correlated gene sets. Specifically, we evaluated the effectiveness of finding individual genes that correlate with pre-defined mitochondrial and lysosomal gene sets and the more broad utility of this approach to associate genes with other cellular components and processes. Of the gene sets examined, those relating to environmental sensing were associated with the largest fraction of the genome. We found implementation of this method to be straightforward and computationally efficient, while often producing meaningful associations consistent with the biological literature. Further, we propose that a variety of secondary analyses that incorporate orthogonal data can be applied to aid in the understanding of biological networks and gene regulation.

## Methods

### Overview

**Figure 1:**
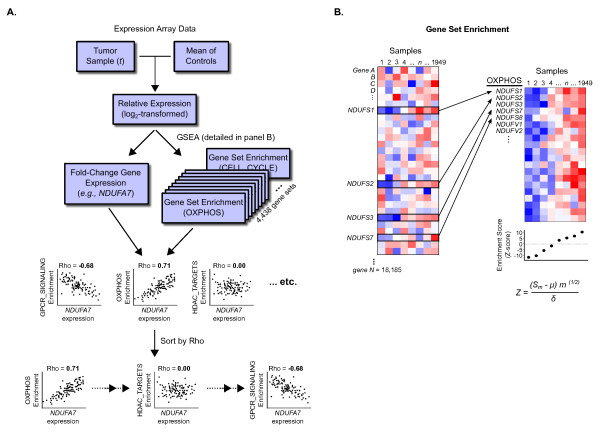
**Schematic for comparison of individual gene expression values to enrichment in gene sets. A**) Relative, log_2_-transformed gene expression array data is computed for each tumor sample *t* (N=1,949) with tissue-matched controls. For each tumor, enrichment in each of the 4,438 gene sets described in Methods is computed. Comparison of fold-change gene expression values with enrichment scores is conducted using Spearman correlation. For a given gene, such as the hypothetical example *NDUFA7*, gene sets are sorted by the correlation coefficients (see Methods for detailed description). **B**) Enrichment in gene sets is calculated using the parametric gene set enrichment approach from Kim and Volsky (2005). Variables used in *Z*-score calculation are described in Methods.

One objective of this study was to determine the extent that the expression of an individual gene was correlated with one or more gene sets. To perform this association, first the overall expression characteristics of a gene set, such as OXPHOS, are collapsed into a summary statistic that captures the magnitude and the variability of expression of genes within the gene set [25]. Second, this measure of enrichment is compared with an individual gene’s fold-change expression value (Figure 1A). Two types of inputs are required to perform this analysis: 1) an *a priori* defined gene set and 2) a gene expression data set that contains sufficient gene expression variability to associate individual genes with the gene set. Data sets with high variability often allow for an increase in the signal to noise inherent in the data. We, like others, noted that large variability in individual gene transcript levels exists in tumor tissue when compared to non-diseased cells isolated from the same tissue type. For example, a 16-fold range of normalized fold-change expression values was found for the nuclear encoded mitochondrial gene *NDUFA7* and many other mitochondrial subunits in the samples from the Expression Project for Oncology data set (expO, Additional file 1: Figure S1). Therefore, we used this tumor tissue-derived expression data set for our analysis. For the *a priori* defined gene sets, we used 4,438 human gene sets included in the MSigDB. 

To implement this approach for a given gene set [27] and a given tumor sample, the expression levels of the genes in the gene set were extracted, transformed to log_2_-space (fold-change), and an enrichment score was produced that summarizes the expression levels of those genes in that particular sample (Figures 1A). The enrichment score in this analysis is the score proposed by Kim and Volsky [25] (Figure 1B) which comprises the average expression value of the genes in the set, weighted by the variability of expression and the number of genes in the set (Z-score). However, other similarly calculated parametric enrichment scores could also be used [28,29]. For a given gene set, this process was repeated for every sample in the expression data series to yield sample-wise enrichment scores. The fold-change expression value for an individual gene was then compared to the gene set enrichment score across all samples using a Spearman correlation coefficient.

### Processing of gene expression datasets

Gene expression data from the human tumor data series of the Expression Project for Oncology (expO) were used and are publicly available from the GEO database (GSE2109). This data series contains gene expression data sets representing 1949 tumor samples of various origins and classifications, conducted with Affymetrix HG-U133 Plus2 arrays. Control samples were chosen from a compendium of array data for non-diseased human tissue, also publicly available from the GEO database (GSE3526, N=163) and Affymetrix [30], N=33). Sample datasets used in the analysis were hand-selected such that the tumor sample data was paired with tissue-matched control data for a total of 1949 tumor samples and 196 controls and only relative log_2_-transformed (fold-change) values were used as expression values in the subsequent analyses ( Additional file 2: Table S1). The data analyses were performed in the R statistical environment v2.11.11 [31,32] with software available from the BioConductor Project (*version 2.5*). Robust Multichip Average (RMA) preprocessing was used for background adjustment, normalization, and summarization of raw expression image intensities, as implemented in the *Affy* package (1.24.2) with updated probeset mappings [33,34].

### Gene set enrichment analysis

Parametric gene set enrichment scores (Z-scores) were computed as implemented in the PGSEA package (version 1.20.1) [25] following standardization of each gene expression value to the median expression value of that gene in tissue-matched controls. Using the formula from Kim and Volsky (2005), the Z score was calculated as *Z = (S*_*m*_-μ) * m^*1/2*^*/ δ*, where *μ* is the mean of fold-change gene expression values from an individual sample data set, *δ* is the standard deviation of the fold-change values from the individual sample data set, *S*_*m*_ is the mean of fold-change values for gene set members in that data set, and *m* is the size of the gene set (Figure 1B). Gene sets were obtained from the Molecular Signatures Database [35] (MSigDB v.3.0 [27], curated from online pathway databases, biomedical literature, positional information, and microarray studies). Gene sets with fewer than 22 or greater than 800 genes were removed to limit biases due to very small and very large gene sets.

### Correlation analysis

Using the preprocessed and standardized expO gene expression data and the matrix of enrichment scores derived from that data (gene sets listed as rows and samples as columns), Spearman correlation was conducted for all combinations of genes measured on the array and gene sets that were used to compute the enrichment scores. This produced an 81 million-element matrix of rho correlation coefficients through the comparison of 18,185 genes and 4,438 gene set enrichment scores (see Additional file 3: Table S2). A correlation statistic was thus found for each possible pair of fold-change expression values and enrichment scores across all tumor samples. To calculate a “relative rank”, the distribution of these correlation statistics was translated to have a mean of 0 and the transformed correlation statistics were ranked in magnitude. In this manner, a strong correlation corresponded to a low relative rank and gene sets associated with each gene were filtered according to the relative ranking. When examining individual gene sets for co-regulated genes, enrichment scores were computed after removing each gene from the gene set. This step was necessary in order to eliminate the possibility of autocorrelation between a gene, *G,* that is a constituent of gene set *S* that might produce an enrichment score heavily influenced by that gene.

### Ranking and filtering results (and relative rank calculation)

To find genes that were associated with a given gene set, genes were ranked by rho correlation coefficients to produce an ordered list of genes whose expression was correlated with the enrichment scores of the gene set across the tumor samples. To find gene sets that were associated with a given gene, a similar process was used. Importantly, gene sets that were directional down (*e.g*., GENESET_EXAMPLE_DN) were grouped as the opposite correlation sign for consistency in interpretation. To gain a measure of confidence in the associations observed compared to the distribution of all possible associations in the data set, we calculated a relative rank for each gene-by-gene set association. The relative rank was computed using the rank of the correlation coefficient for the gene with each gene set compared to the distribution of all possible correlation coefficients (Additional file 1: Figure S2). The lists of gene sets associated with each gene were thus sorted by the relative ranks. Further, gene sets such as certain cancer “modules” were left out of the rankings if they were not clearly interpretable [14,36]. Not all genes described in the book *Genome* were examined nor were all of the chromosomes represented. 5SRNA genes were described for chromosome 1 and *NROB1* for the X chromosome, but were not measured on the array. Genes were also not explicitly provided for chromosomes 2, 21, and 22.

### GO annotation enrichment

To further test whether genes whose expression was found to be correlated with enrichment in the REACTOME_OLFACTORY_SIGNALING_PATHWAY and the SHEN_SMARCA2_TARGETS_DN gene sets were related to olfactory signaling and environmental sensing, we computed and ranked posterior probabilities for enrichment in GO terms. The posterior probabilities were calculated after controlling for multiple testing and using all GO categories with greater than five members, as implemented in the MGSA package [37]. Gene sets with posterior probabilities greater than 0.50 were regarded as active, as suggested by Bauer *et al.* (2011).

## Results

**Figure 2:**
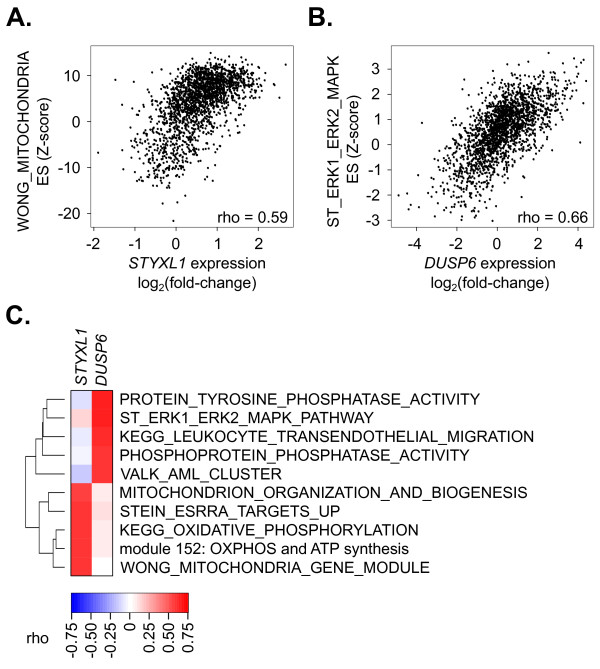
**Correlation of a fold-change gene expression value with a gene set-derived enrichment statistic can elucidate the function of poorly characterized gene products. A**) Plot of fold-change gene expression values for the gene *STYXL1* versus the enrichment statistics calculated using the WONG_MITOCHONDRIA gene set. Data points are derived from individual samples within the expO data series (see Methods). **B**) Plot of the fold-change expression values for *DUSP6* versus enrichment statistics derived from the gene set ST_ERK1_ERK2_MAPK_PATHWAY. **C**) Heatmap of correlation statistics for the five gene sets that were most strongly associated with *STYXL1* or *DUSP6*, as determined from all gene sets examined (N=4,438). The dendrogram on the left represents the overlap between the gene sets. The dendrogram was generated using dissimilarities between gene set compositions as previously described [73].

The approach is demonstrated using the *STYXL1* gene. Following a siRNA screen, several genes were identified that enhanced or diminished the sensitivity of cells to chemotherapeutics [38]. One of these genes, *STYXL1*, was a prominent hit in the siRNA screen but the cellular function of this gene was largely unknown. *STYXL1* shows homology to protein phosphatases but lacks a critical cysteine residue that is deemed essential for catalytic activity [39,40]. To identify potential molecular functions of *STYXL1*, the expression of *STYXL1* was compared with gene set enrichment scores derived from all possible gene sets to discover which gene sets were most closely associated with *STYXL1*. Statistical methods that compute significance values corrected for multiple testing are difficult to apply to this analysis due to the large number of overlapping, non-independent gene sets that violate assumptions of independence. Rather than account for the overlap when determining the confidence [41], we noticed that the population of rho coefficients were symmetrically distributed with a mean of −0.0262 and a standard deviation of 0.261 (Additional file 1: Figure S2). We leveraged this finding to approximate our confidence in the gene-gene set associations with relative ranks of individual gene-pathway rho coefficients (both positive and negative) by first centering the distribution on zero and then ranking the absolute values of individual gene-pathway rho coefficients. The gene set that correlated most strongly with *STYXL1* transcript levels was known to be mitochondrial-related, WONG_MITOCHONDRIA (Additional file 3: Table S2, Figure 2A). The association of *STYXL1* with the gene set WONG_MITOCHONDRIA had a correlation coefficient equal to 0.59 that ranked 879,685^th^ of the 80,705,030 coefficients calculated. This produced a relative rank of 0.011, indicating the rho correlation coefficient obtained from this gene-gene set pair was in the top 1.1% of all gene-gene set pairs examined. Further, among the compendium of gene sets tested, several of the strongest correlations with *STYXL1* transcript expression were mitochondrial gene sets (Figure 2C). In support of this association, *STYXL1* was shown to be a previously uncharacterized mitochondrial protein [42]. We also examined potential roles of *DUSP6*, a protein phosphatase that shows high homology with *STYXL1* but contains the cysteine residue required for catalytic activity. In contrast to *STYXL1*, *DUSP6* was most strongly correlated with phosphatase-related gene sets, such as ST_ERK1_ERK2_MAPK (Figure 2B and C). The association of *DUSP6* with the ST_ERK1_ERK2_MAPK gene set was assigned a relative rank of 0.004, indicating that this gene-gene set pair were in the top 0.4% of all gene-gene set pairings. 

To determine if similar gene set associations were evident for *STYXL1* and *DUSP6* using gene-network model approaches, we input the *STYXL1* and *DUSP6* gene names into the online tools GeneMANIA [43] and COXPRESdb [44]. The GeneMANIA tool uses available evidence from physical interactions, co-expression, predictions, pathways, co-localization, shared protein domains, and genetic interactions to create a network of genes related to the gene(s) input into the model [45] while the COXPRESdb tool uses coexpression of genes across seven animal species to build gene networks [46]. We noted that explicit pathway associations were not available for *STYXL1* using these tools. Thus, to determine pathway associations for these two genes, we extracted the 20 most significantly associated genes using each tool and compared them to the same compendium of gene sets described above, ranking according to significance with a hypergeometric distribution. Using GeneMANIA, the gene sets most related to *STYXL1* were KEGG_PEROXISOME (p=0.003) and RICKMAN_METASTASIS_UP (p=0.004). In contrast to the frequent mitochondrial gene set associations (18 of the top 20 correlating gene sets) when using the gene expression and enrichment score correlations (Figure.2A and 2C), only one mitochondrial gene set appeared in the 20 most strongly associated gene sets using this gene-network model. When using COXPRESdb, the gene sets most related to *STYXL1* were found to be chr7q22 (p<0.001) and MORF_XPC (p<0.001). Three mitochondrial gene sets were evident in the 20 most-significantly associated gene sets with this model. Both of the models showed a variety of gene sets related to *DUSP6*- primarily MAPK signaling, growth factor signaling, and phosphorylation activity.

**Figure 3:**
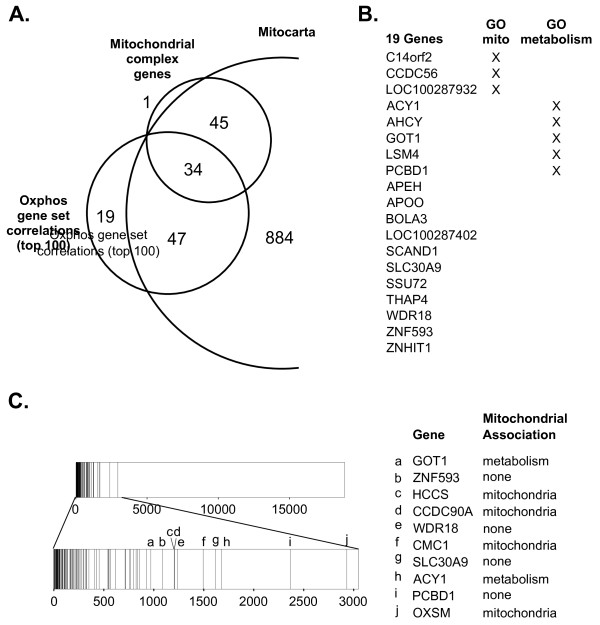
**Genes that associate most strongly with the OXPHOS expression phenotype are mitochondria-associated. A**) The 100 most strongly correlated genes with the MOOTHA_VOXPHOS gene set (OXPHOS gene set correlations) as compared to known electron transport subunit genes (OXPHOS genes, see Main Text) and mitochondrial-associated genes from the Mitocarta compendium. Genes were filtered to only include those that were interrogated in the gene expression array data. Nineteen genes associated with MOOTHA_VOXPHOS were not associated with Mitocarta or known mitochondrial subunit genes. **B**) The Gene Ontology classifications of the 19 OXPHOS gene set correlation genes described in A. **C**) Rank plot showing the top 100 OXPHOS-correlated genes described in A versus the predictions made by Baughman *et al.*[48]. Also shown are the GO associations of 10 of the 100 OXPHOS-correlated genes that were ranked lower by Baughman et al (a-j).

Proteins located within the mitochondria play an important role in cellular metabolism through the process of oxidative phosphorylation (OXPHOS). OXPHOS is accomplished by a series of protein complexes within the mitochondria known as complexes I, II, III, IV, and V, encoded by approximately 100 genes. Several reports show that genes whose protein products are located in the mitochondria tend to be transcriptionally co-regulated [5,9,47-49]. Therefore, to test whether this gene – gene set association existed for genes encoding complexes I-V, we calculated enrichment in the expression of known nuclear-encoded OXPHOS related genes, given by the MOOTHA_VOXPHOS gene set [49], and compared the enrichment scores to fold-change gene expression values for individual genes encoding complexes I-V. We found enrichment values obtained using the MOOTHA_VOXPHOS gene set were correlated with the expression of individual OXPHOS mitochondrial subunit genes in the samples of the expO tumor dataset. Ninety percent (72/80) of the OXPHOS mitochondrial subunit genes were correlated with enrichment in the MOOTHA_VOXPHOS gene set with a relative rank of less than 0.05 (corresponding to a rho of greater than 0.50, Additional file 1: Figure S3). Though known OXPHOS mitochondrial subunit genes correlated with the MOOTHA_VOXPHOS gene set, we also determined which genes showed the strongest correlations with the MOOTHA_VOXPHOS gene set. Upon examining the 100 genes with the largest rho correlation coefficients, we found that 81 were known to be either OXPHOS mitochondrial subunit genes or in the mitochondrial protein compendium, Mitocarta (Figure 3A). Note that the total number of Mitocarta genes (N=1010) is less than the total number of genes in the database (N=1098) because not all of the Mitocarta genes were represented in the gene expression array data. Nineteen of the genes whose expression was strongly correlated with the OXPHOS gene set were not in Mitocarta, indicating that there is not current evidence showing strong mitochondrial localization of their protein products. However, eight of these genes have GO annotations relating to mitochondria or metabolism, suggesting they may be functionally related to OXPHOS (Figure 3B). 

In addition, we compared the 100 genes with the highest rho correlation coefficients using our method to the scores generated by Baughman *et al.*[48] who calculated the probability that a gene is co-expressed with the OXPHOS gene set (Figure 3C). The method proposed by Baughman *et al.* requires the curation and downloading of 1,427 individual human and mouse gene expression datasets, the preprocessing of each data set, calculation of correlation within each dataset, and combination of the resulting correlations to build a weighted summary score. The result of this activity is a single gene-gene set pair score. Our approach is comparatively simple, using only the human expO microarray data set and tissue-matched normal data. The initial findings from our approach were qualitatively similar to those obtained using the computationally intensive approach of Baughman *et al.* The top 100 genes we found strongly correlated with the MOOTHA_VOXPHOS gene set were also ranked highly in the Baughman *et al.* list of OXPHOS probability scores for each gene. Conversely, comparison of the top 100 genes identified in the Baughman *et al.* list showed high rankings within our ranked list of all genes in the datasets (Additional file 1: Figure S4).

In order to test this model beyond mitochondria, we assessed the co-regulation of individual lysosomal genes with enrichment in the lysosome gene set, KEGG_LYSOSOME. Lysosomal genes also show a high degree of transcriptional co-regulation in tissue culture cells (Sardiello et al 2009). The 50 genes that correlated strongest with this gene set were identified and compared with genes that were either identified as being lysosomal via a proteomic survey or associated with lysosomal GO annotation (Accession GO:0005764) [50]. Fifty percent (25/50) of genes identified encode for lysosome-associated proteins (Additional file 1: Figure S5A). Of the twenty-five genes associated with the KEGG_LYSOSOME gene set that were not in the proteomic survey or identified with the lysosomal GO annotation, 19 (76%) possessed GO terms related to the endoplasmic reticulum, golgi, or vacuoles (Additional file 1: Figure S5B). Therefore, 88% (44/50) of genes associated with the KEGG_LYSOME gene set were related to vesicle transport.

**Figure 4:**
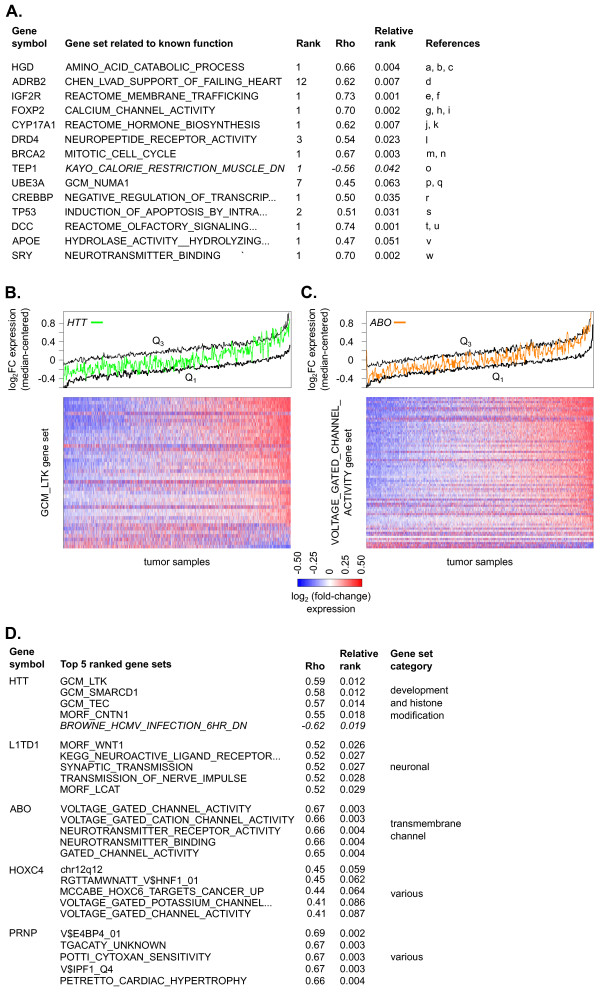
**Enrichment scores of gene sets are related to the gene’s known biological role. A**) The ranks of the strongest-correlated gene sets with similar characteristics to the known biological roles of genes highlighted in the book *Genome*. References are listed below. **B**) Plot of the *HTT* gene expression profile across all samples (green line, running median window size=11) compared to the median-centered fold-change expression for all genes of the most strongly correlated gene set, *GCM_LTK* (black lines: Q1= first quartile, Q3 = third quartile, heatmap: N=41). **C**) Plot of the *ABO* gene expression profile across all samples (orange line, running median window size=11), compared to the median-centered fold-change expression for all genes of the most strongly correlated gene set, VOLTAGE_GATED_CHANNEL_ACTIVITY (black lines: Q1= first quartile, Q3= third quartile, heatmap: N= 72). **D**) Genes from the book *Genome* with poorly- or broadly-characterized roles and the top five ranking gene sets by correlation coefficients for each gene are listed. In A and D, gene sets were listed according to their relative ranking (i.e. distance from the mean of the null distribution) and “DN” gene sets with negative correlations are shown in italics. Uninformative cancer gene modules were not included. References for part A: [79]^a^, [80]^b^, [81]^c^, [82]^d^, [83]^e^, [84]^f^, [85]^g^, [86]^h^, [87]^I^, [88]^j^, [89]^k^, [90]^l^, [91]^m^, [92]^n^, [93]^o^, [94]^p^, [95]^q^, [96]^r^, [97]^s^, [98]^t^, [99]^u^, [52]^v^, [100]^w^.

To examine the effectiveness of this method for insight into protein functions more broadly, we examined a set of genes with widely recognized biomedical significance, described in the popular biomedical science book *Genome,* by Matt Ridley [51]. In this book, Ridley describes a set of well-studied genes that lie on different chromosomes. For each of these genes, we found the highest ranked gene set representative of its role based on curation of the literature. Note that this was not meant to represent an exhaustive summary of the literature but instead a sample of studies or reviews suggesting the identified relationships. Of the 14 well-studied genes described, representative gene sets were found within the top five ranked correlations for 12 of the genes (Figure 4A). In all cases (14 of 14), the relative ranks for the correlations were in the top 10 % of all correlation coefficients (*i.e.* less than 0.10). Expression of the breast cancer susceptibility gene, *BRCA2*, was most strongly correlated with gene sets representing cell cycle checkpoints and DNA damage response (Figure 4A and Additional file 3: Table S2). These gene sets are consistent with the role for *BRCA2* in *DNA* repair and cell cycle checkpoints that has been well established in the literature. Expression of the apoprotein gene *APOE* correlated with gene sets related to hydrolase and peptidase activity as well as lipid metabolism. These gene sets are also consistent with the known role of APOE as a mediator of normal lipid and amino acid metabolism [52,53]. *FOXP2* is a gene encoding a transcription factor that is strongly tied to an autosomal dominant speech and language neurological disorder [54]. A binding partner *to FOXP2, FOXP1*, has recently been found confined to projection neurons [55]. Though the specific function of *FOXP2* in neurons remains to be elucidated, expression of *FOXP2* corresponded best with enrichment in a gene set related to neurotransmitter release, CALCIUM_CHANNEL_ACTIVITY. Other highly correlated gene sets included additional neuron-related gene sets as well as gene neighborhoods, consistent with its role as a transcription factor (Additional file 3: Table S2). 

Five genes shown in the list- *HTT, L1TD1*, *ABO*, *HOXC4*, and *PRNP*, have poorly defined or ambiguous biological roles. To determine whether these genes associate strongly with any gene sets, we examined the top ranked gene sets (Figure 4B-D). For the huntingtin gene, *HTT*, an association was found with the brain specific kinase LTK (Figure 4B and 4D). This association does not appear to be driven by a single gene since most of the genes of the LTK gene set show a similar trend in expression as *HTT* across the tumor samples (Figure 4D). Though there is evidence that LTK has a role in apoptosis [56], additional gene sets appear to be related to immune function and development pathways for which there is also evidence *HTT* is involved [57-59]. Expression of the gene *ABO* was strongly correlated with several gene sets related to the activity of transmembrane channels, indicating a potential role of this gene (Figure 4C and 4D). For three genes, *L1TD1*, *HOXC4*, and *PRNP*, a single biological role is not apparent in this analysis. In summary, in 16 of 19 (84%) cases, the predictions generated by the integration of fold-change gene expression and gene set enrichment scores were associated with consistent biological processes (Additional file 3: Table S2).

**Figure 5:**
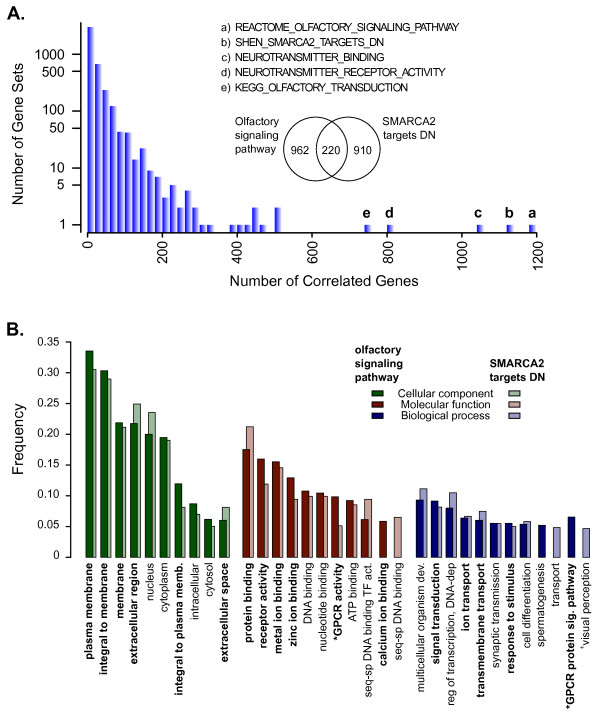
**Environment sensing gene sets are associated with the largest numbers of genes. A**) Histogram of the number of gene sets associated with a given number of genes. The frequency of the gene set was based on how often the gene set appeared in the top five correlated gene sets for each gene. The Y-axis is log_10_-transformed. Inset is a Venn diagram showing the degree of overlap between the genes associated with the gene sets REACTOME_OLFACTORY_SIGNALING_PATHWAY and SHEN_SMARCA2_TARGETS_DN. **B**) Histogram showing the 10 most frequently represented GO pathways for genes associated with, but not members of, the gene sets REACTOME_OLFACTORY_SIGNALING_PATHWAY (dark colored bars) and SHEN_SMARCA2_TARGETS_DN (light colored bars). In bold are GO terms most closely related to environmental sensing. GO annotations are grouped into categories: cellular component (green), molecular function (red), and biological process (blue). Frequencies are based on the number of genes with a specific GO annotation versus the total number with GO Annotations (totals for REACTOME_OLFACTORY_SIGNALING_PATHWAY: CC, N=734; MF, N=642; BP, N=607; totals for SHEN_SMARCA2_TARGETS_DN: CC, N=705; MF, N=637; BP, N=596). Significant enrichment in GO categories was found for the olfactory* and Smarca2^+^ associated genes using the model-based gene set analysis (MGSA) approach, as described in the Methods.

While examining the gene set associations for these and other genes, we noticed that some gene sets seemed to be more frequently associated with genes than others. To determine which gene sets were related to the largest repertoire of genes, we examined the five strongest correlating gene sets for every well-measured gene in the expression dataset. The largest number of genes was correlated with enrichment in gene sets related to chemo-sensing (amino acid and small odorant receptors) and transmembrane channel signaling (Figure 5A). Specifically, a gene set composed of potential odorant receptors, REACTOME_OLFACTORY_SIGNALING_PATHWAY, was correlated with over 4% of the genes in the dataset. Olfactory receptors are G-protein coupled receptors expressed in the main olfactory epithelia and other tissues that bind small, volatile 'odorous' molecules [60,61]. The olfactory gene family is the largest family of genes with more than a thousand genes encoding potential odorant receptors in humans [60,62]. However, of the 1,182 genes we found co-regulated with genes of the REACTOME_OLFACTORY_SIGNALING_PATHWAY, only 47 were constituent genes of the gene set (4%), suggestive that additional genes may play a role in this process. 

The olfactory receptor protein family consists of mainly seven-transmembrane receptors that are linked to ion flux through the cAMP-signaling pathway. Binding of small molecules to the receptor induces a signaling cascade whereby the activated receptor activates a G-protein, leading to the production of adenylyl cyclase. Subsequently, adenylyl cyclase binds to a cyclic-nucleotide-gated-ion channel, inducing channel opening and depolarization of the cell membrane. To determine whether the genes co-regulated with the REACTOME_OLFACTORY_SIGNALING_PATHWAY gene set were involved in the olfactory signaling process, we placed the genes with GO annotations into groups based on those annotations (Figure 5B). Consistent with this signaling cascade, the genes that were not members of the gene set could be classified predominantly to membrane signaling with activities related to GPCR activity, ion transport, ATP-binding, and signal transduction. Interestingly, many of the genes associated with the REACTOME_OLFACTORY_SIGNALING_PATHWAY gene set were uncharacterized proteins or open reading frames (196/1182 16.6%). The association with this number of uncharacterized proteins is nearly double what would be expected by chance (p<0.00001).

The second most prevalent gene set association involved SHEN_SMARCA2_TARGETS_DN related genes (Figure 5A). Although the two gene sets SHEN_SMARCA2_TARGETS_DN and REACTOME_OLFACTORY_SIGNALING_PATHWAY had little overlap (N=74, N=327, respectively; overlap=3 genes), the genes whose expression was correlated with enrichment in these gene sets were somewhat redundant (Venn diagram, Figures 3 and 4A). SMARCA2 is a component of the large SWI/SNF ATP-dependent chromatin-remodeling complex required for transcriptional activation of genes repressed by chromatin. Similar to the olfactory signaling gene set, of the 1,130 genes whose expression correlated best with SHEN_SMARCA2_TARGETS_DN, only 99 genes were constituent members of the gene set (9%). Also, like the olfactory signaling gene set, the associated non-constituent genes could be primarily classified to membrane signaling, GPCR activities, ion transport, ATP-binding, and signal transduction GO and PFAM annotations (Figure 5B). As with the REACTOME_OLFACTORY_SIGNALING_PATHWAY, more of the genes associated with SHEN_SMARCA2_TARGETS_DN coded for uncharacterized proteins than would be expected by chance (162/1130, 14.3%, p<0.00001).

We examined the genomic positions of the genes associated with these two gene sets to determine if shared cis-regulatory elements might exist between the correlated genes and the constituent genes of the gene sets. However, neither the genes co-regulated with REACTOME_OLFACTORY_SIGNALING_PATHWAY nor the genes co-regulated with SHEN_SMARCA2_TARGETS_DN were consistently mapped near the constituent genes of the respective gene sets (p=0.49 and p=0.66, respectively). The SHEN_SMARCA2_TARGETS_DN gene set was constructed by examining genes that correlated inversely with *SMARCA2* expression in prostate cancer samples [63]. The association of this gene set with olfactory signaling indicates a potential link between chromatin remodeling and the expression of chemo-sensing genes as recently described by Magklara *et al.*[64].

## Discussion

We showed that correlation of fold-change gene expression values and gene set enrichment scores is an effective method of inferring cellular roles of genes. Using this correlation procedure has allowed us to confirm known roles for well-characterized genes by ranking the correlations of the gene expression with a compendium of gene sets. The approach described is computationally efficient and simple to implement and we demonstrated that its ability to identify mitochondria-related genes is as effective as approaches recently devised by Baughman *et al.*[48]. Notably, the association matrix took approximately 57 hours of computational time to generate using a 2.2GHz (8-cores) processor with 9.6 Gb of RAM. Therefore, this type of association matrix can be computed from a variety of gene expression datasets using a variety of gene sets. Likewise, we have also implicated a mitochondrial role for several genes not currently known to be associated with mitochondria through the “guilt-by-association” paradigm. Following this paradigm, we found that a variety of individual genes were associated with gene sets functionally related to the known roles of those genes and that genes with unknown roles tended to be associated with gene sets that represent biological themes. We have generated a catalog of co-regulated gene sets (CGs) that includes all 18,185 genes in the dataset (Additional file 3: Table S2). This has the potential to inform a variety of high throughput genomic approaches.

Although olfactory receptors are in a large family of G-protein coupled receptors (GPCRs), the association of many other genes with the REACTOME_OLFACTORY_SIGNALING_PATHWAY gene set was somewhat surprising. Olfactory receptors are commonly associated with environmental sensing, including the perception of tastes, odors, and chemical moieties through specialized sensory receptor cells in the nose, mouth, tongue, and skin. Information about a stimulus is transduced through a cascade of molecular and cellular events into electrical signals that are recognized by the nervous system. However, recent reports indicate that olfactory receptors may play a much more diverse role in chemosensing [65-68]. Epithelial cells along the entire gastrointestinal tract are involved in chemosensing and proposed nutrient absorption [68]. Outside of the gastrointestinal epithelium, olfactory receptors have been found to play important roles in myogenesis, and at least one receptor, MOR32, could direct myocyte migration through a yet to be identified soluble chemo-attractant secreted by fusing muscle cultures [69]. Multiple studies also indicate the importance of olfactory receptors in sperm cell chemotaxis [70,71]. Perhaps most interestingly, an olfactory receptor over-expressed in prostate cancer, *OR51E2*, interacts with androstenone derivatives and this interaction regulates cell proliferation [72]. These findings suggest that chemosensing mechanisms operate in diverse cell types. Our results support this possibility and implicate a significant fraction of the coding genome in support of this type of signal transduction cascade. Several somatic mutations that have been identified in cancer cells are associated with olfactory signal transduction using the method put forth in this report (Additional file 3: Table S2). Whether these mutations lead to disrupted signal transduction important to tumorigenesis or proliferation of cancer cells remains to be explored.

As with all computationally-derived guilt-by-association correlations, we also point out limitations to this type of analysis which include 1) the trends may be dependent on the expression data used, 2) the analysis may be affected by low quality gene sets or gene sets with ambiguous biological underpinnings, and 3) the enrichment statistic can be influenced by expression of a gene if it resides within the actual gene set (*i.e.* self-fulfilling associations). However, in the latter case the calculation used for the enrichment statistic should at least partially compensate for this effect. Though we used tissue-matched normal tissues to standardize the tumor tissues in our analysis, we also conducted similar analyses using only normal tissues and using only tumor tissues. Many of the gene expression-by-gene set enrichment correlations were similar to our initial findings when we used these smaller, median-standardized subsets of expression data. However, there tended to be less expression variability for a given gene across the restricted data sets and genes with low expression variability tended to produce incorrect gene set associations. We also noted this effect when using a smaller set of tumor-normal gene expression data as described in [73]. Perhaps intuitively, a sample set with sufficient expression variability in the gene of interest should be used. Two obvious sources of expression variability may come from using a greater number of samples or increasing the heterogeneity of the tissue types used.

The general effectiveness of computationally-derived guilt-by-association approaches is difficult to evaluate. Without detailed *in vitro* and *in vivo* experimental manipulations and follow-up studies, genes used for validation of an association must be supported by a large body of literature explicitly detailing both their molecular functions and biological roles. Some of the best-studied genes are those with strong links to human health and disease, as described in the book *Genome.* Though we examined these genes with our guilt-by-association model, it is possible that biomedically prominent genes are biased for their pleiotropic effects and the model might be less effective for genes with more subtle roles. Further, interpretation of the literature can be subjective, especially with the burgeoning numbers of publications and bias in not publishing “negative” data. In spite of these challenges, guilt-by-association models have served to inform a variety of hypotheses over the past two decades and have been extended in this report with the generation of sample-wise enrichment scores.

In addition to guilt-by-association, the generation of sample-wise enrichment scores for numerous, diverse gene sets lays the foundation for analyses similar to those recently devised for gene expression array data. Data mining approaches that are commonly used for gene expression analysis (clustering approaches [74], discriminate analysis [75], and outlier analysis [76]) can be applied at the level of pathways/gene sets and can be integrated with other types of orthogonal data sets such as genotype information (*e.g*. “ping-pong” analysis [77]). We have used a variation of outlier profile analysis (meta-COPA [78]) to identify tumor samples in this dataset that contained a gene signature indicative of activation of the NRF2 transcription factor. The identification of subsets of samples that share a similar biological activity, such as NRF2 activity, can assist in the identification of disease subgroups that may not be well described or may respond differently to treatments. Several other gene set enrichment approaches require that the samples of interest be partitioned into *a priori* defined groups for the application of the enrichment analysis. Unless a signal transduction transcriptional phenotype is stronger than other transcriptional effects related to the *a prior* group assignments (such as tissue type or proliferation rate), more subtle pathophysiological transcriptional effects can be overwhelmed and consequently overlooked with those traditional approaches.

## Conclusions

We have outlined a computational approach that has proven useful to predict gene function using transcriptional data. The approach we describe was successfully used to identify molecular functions of genes that were previously not well characterized [42,73]. We found that gene transcripts that correlated best with enrichment in mitochondrial and lysosomal gene sets often possessed mitochondrial and lysosomal roles as evidenced in the literature. Furthermore, gene sets reflective of a gene’s known cellular role were more strongly correlated with the gene’s expression than unrelated gene sets. Using this method, we noted the high number of genes whose transcripts correlated strongly with enrichment in gene sets representing small molecule and small peptide signal transduction. Our results show that this approach is useful to infer functions of disparate sets of genes with prominent roles in the biomedical literature and may complement current methods in situations where other databases are unable to provide predictions of gene functions. Further, the effectiveness, versatility, and breadth of this approach make possible its application across a variety of contexts and with a variety of downstream analyses.

## Availability and requirements

**Project name:** Genome-wide matching of genes to cellular roles using guilt-by-association models derived from single sample analysis

**Project home page:** NA

**Operating system(s):** platform independent

**Programming language:** NA

**Other requirements:** R, Bioconductor

**License:** NA

**Any restrictions to use by non-academics:** NA

## Availability of supporting data

All data is from publicly available sources (refer to Additional file 2: Table S1).

## Competing interests

The authors note that they have no competing interests to declare.

## Authors’ contributions

JK performed the data analyses, JK and KF contributed to the study design and manuscript preparation. All authors read and approved the final manuscript.

## Supplementary Material

Additional file 1: Figure S1 High gene expression variability exists for nuclear encoded mitochondrial complex genes in the expO tumor data set. A) Log_2_-transformed expression of the mitochondrial gene *NDUFA7* in 1949 samples of the expO data set with summarization whisker plot. B) Summarization whisker plots for all nuclear encoded mitochondrial complex genes measured on the gene expression array, sorted by median fold-change expression in tumors relative to normal samples. **Figure S2.** Distribution of rho correlation statistics for all gene expression-by-gene set enrichment pair-wise comparisons (N=8.07x10^7^). **Figure S3.** Genes encoding mitochondrial complex genes are correlated with enrichment in an OXPHOS gene set. Nuclear encoded mitochondrial complex genes (N=80) are sorted by their rho correlation coefficients calculated by comparing with enrichment in the MOOTHA_VOXPHOS gene set across tumor samples of the expO data set. **Figure S4.** Genes predicted to be strongly associated with OXPHOS using the method by Baughman *et al.* (2009) are also ranked highly in the list of genes correlated with the MOOTHA_VOXPHOS gene set. Rank plot showing a comparison of the top 100 genes predicted to be associated with OXPHOS by Baughman *et al.* (2009) compared to the rank of the genes using our method. The 10 most poorly ranked genes are listed as a-j with indication of whether they possess a mitochondria or metabolism related GO association. **Figure S5.** A lysosome gene expression phenotype is associated with changes in the expression of constituent genes and genes with known roles in lysosomes and related processes. A) Venn diagram showing 50 genes that correlate most strongly with theKEGG_LYSOSOME gene set compared to known lysosomal genes (associated with the GO term “Lysosome” as well as those identified in Lubke *et al*. (2009)). B) Listing of the 50 genes that correlated best with the lysosome gene set from A. In the first column are known lysosomal genes, in the second column are genes not known to be associated with lysosomes, and the third column lists examples of GO terms associated with the non-lysosomal genes. Click here for file

Additional file 2: Table S1 **Catalog of Co-regulated Gene sets (CGs).** Table showing top 10 positive and top 5 negative associated gene sets for all genes measured on the arrays. Note, gene sets were sorted by relative ranks for each gene and “DN” directional gene sets were grouped with the opposite of their rho correlation coefficients (see Methods). Click here for file

Additional file 3: Table S2 List of gene expression data set accession numbers for data used in the analyses. Click here for file

## Acknowldegements

We gratefully acknowledge the Van Andel Research Institute and the Van Andel Education Institute for supporting these studies.
