# Supplementary material for: Genome-wide matching of genes to cellular roles using guilt-by-association models derived from single sample analysis
Source: BMC Res Notes. 2012 Jul 23;5:370. doi: 10.1186/1756-0500-5-370 (PMC3599284; doi:10.1186/1756-0500-5-370)
Supplement: Additional file 1: Figure S1 — High gene expression variability exists for nuclear encoded mitochondrial complex genes in the expO tumor data set. A) Log2-transformed expression of the mitochondrial gene NDUFA7 in 1949 samples of the expO data set with summarization whisker plot. B) Summarization whisker plots for all nuclear encoded mitochondrial complex genes measured on the gene expression array, sorted by median fold-change expression in tumors relative to normal samples. Figure S2. Distribution of rho correlation statistics for all gene expression-by-gene set enrichment pair-wise comparisons (N=8.07x10^7^). Figure S3. Genes encoding mitochondrial complex genes are correlated with enrichment in an OXPHOS gene set. Nuclear encoded mitochondrial complex genes (N=80) are sorted by their rho correlation coefficients calculated by comparing with enrichment in the MOOTHA_VOXPHOS gene set across tumor samples of the expO data set. Figure S4. Genes predicted to be strongly associated with OXPHOS using the method by Baughman et al. (2009) are also ranked highly in the list of genes correlated with the MOOTHA_VOXPHOS gene set. Rank plot showing a comparison of the top 100 genes predicted to be associated with OXPHOS by Baughman et al. (2009) compared to the rank of the genes using our method. The 10 most poorly ranked genes are listed as a-j with indication of whether they possess a mitochondria or metabolism related GO association. Figure S5. A lysosome gene expression phenotype is associated with changes in the expression of constituent genes and genes with known roles in lysosomes and related processes. A) Venn diagram showing 50 genes that correlate most strongly with theKEGG_LYSOSOME gene set compared to known lysosomal genes (associated with the GO term “Lysosome” as well as those identified in Lubke et al. (2009)). B) Listing of the 50 genes that correlated best with the lysosome gene set from A. In the first column are known lysosomal genes, in the second column are genes not known to be associat [file 1756-0500-5-370-S1.pdf]

A. B.

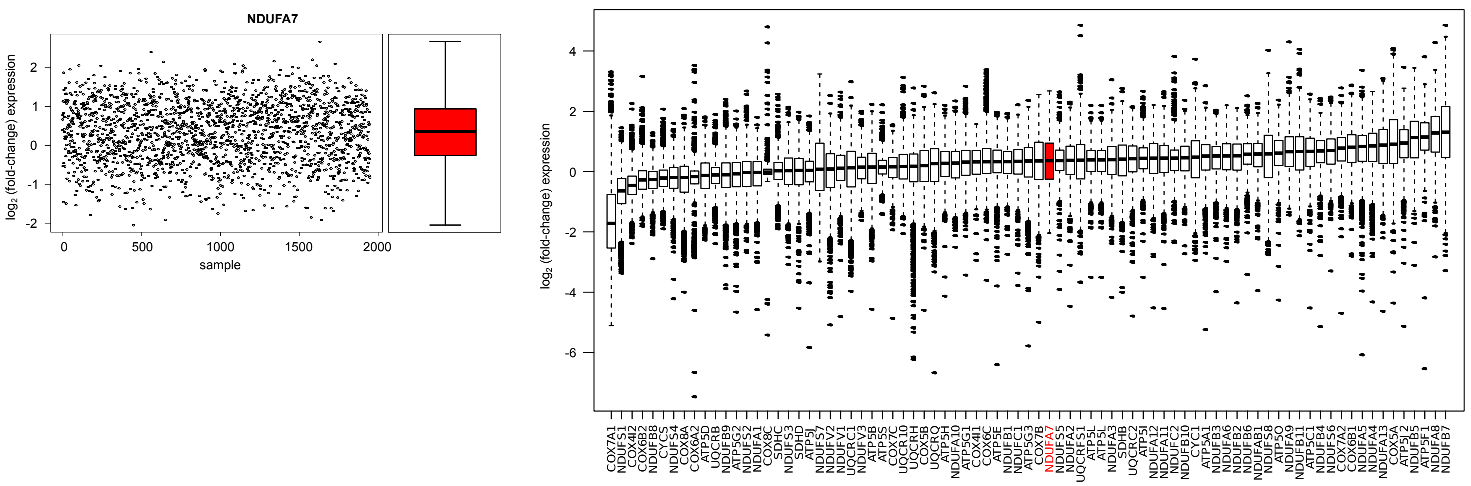

Supplemental Figure 1

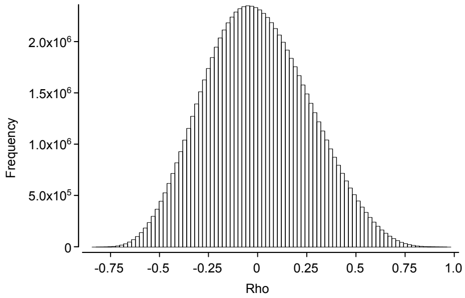

Supplemental Figure 2

Gene	Rho	Gene	Rho	Gene	Rho
COX7B	0.87	NDUFV2	0.75	NDUFB3	0.63
NDUFA13	0.86	NDUFB10	0.75	ATP5E	0.62
NDUFS3	0.85	NDUFA6	0.75	ATP5I	0.62
COX5B	0.85	NDUFA11	0.75	UQCRH	0.61
UQCRC1	0.85	NDUFS7	0.75	NDUFV3	0.60
ATP5B	0.84	COX5A	0.74	SDHD	0.60
ATP5G3	0.83	NDUFV1	0.74	NDUFB1	0.60
UQCRFS1	0.83	NDUFA12	0.74	CYCS	0.59
NDUFS8	0.83	CYC1	0.73	NDUFS1	0.57
COX6B1	0.83	NDUFA9	0.72	SDHC	0.56
NDUFB7	0.82	ATP5J	0.71	UQCRB	0.56
NDUFA7	0.82	COX7C	0.71	NDUFB9	0.56
NDUFA8	0.81	ATP5F1	0.70	NDUFA5	0.54
UQCRQ	0.81	NDUFA4	0.70	NDUFC2	0.53
NDUFB11	0.81	NDUFS6	0.70	ATP5G2	0.53
SDHB	0.80	ATP5A1	0.69	NDUFS5	0.53
NDUFB5	0.80	ATP5D	0.68	NDUFS2	0.51
NDUFA1	0.79	NDUFA10	0.68	ATP5G1	0.50
ATP5C1	0.79	COX7A2	0.68	COX6C	0.45
ATP5J2	0.78	UQCRC2	0.68	ATP5S	0.43
ATP5H	0.77	UQCR10	0.68	NDUFS4	0.34
NDUFA2	0.77	ATP5L	0.66	COX6A2	0.20
ATP5O	0.77	COX4I1	0.66	COX7A1	-0.09
COX8A	0.77	NDUFC1	0.66	COX8C	-0.09
NDUFAB1	0.77	NDUFB2	0.65	COX4I2	-0.37
NDUFA3	0.76	NDUFB6	0.65	COX6B2	-0.44
NDUFB4	0.75	NDUFB8	0.63		

Supplemental Figure 3

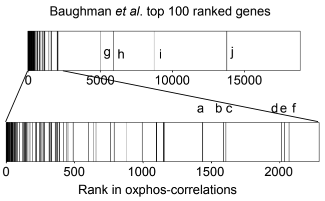

	Gene	Mitochondrial Association
a	MCTS1	none
b	HINT1	none
c	RBX1	none
d	COX6C	mitochondria
e	UQCR11	mitochondria
f	CHCHD2	none
g	TCEB2	none
h	MRPS21	mitochondria
i	RPL24	none
j	C16orf65	none

Supplemental Figure 4

A.

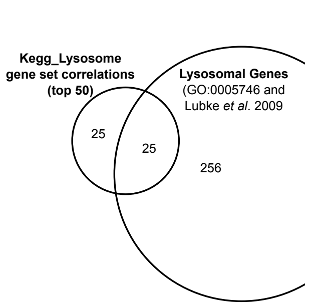

B.

25 lysosomal genes	25 non-lysosomal genes	Lysosome-related GO annotation
ACP5	ATOX1	ion transport
ARL8B	BCAP31	golgi, ER, membrane
ASAH1	C22orf25	NA
ATP6AP1	CAPG	melanosome
ATP6V0B	FTL	golgi, membrane, ion transport
ATP6V0C	GCH1	hydrolase activity, vesicle
ATP6V0D1	GRN	**none**
ATP6V0E1	MFSD1	membrane
ATP6V1B2	MYL12B	**none**
ATP6V1D	NPL	**none**
C1orf85	OAZ1	**none**
CTSA	PLEKHB2	membrane
CTSB	PPP1CA	metabolism
CTSD	PRKCD	ER, membrane, ATP binding
CTSH	RNASEK	hydrolase activity, membrane
GLB1	RNH1	**none**
HEXA	SLC38A6	ion transport, membrane
IFI30	SLC7A7	ion transport, membrane
IGF2R	SPPL2A	peptidase, membrane
LAMP1	TMBIM6	ER, membrane
LAMP2	TMEM9B	membrane
LGMN	TRAPPC5	golgi, ER, vesicle-mediated trans.
NAGA	UBE2A	ATP binding
NEU1	UXS1	golgi, membrane, metabolism
PSAP	VAMP8	golgi, endosome, membrane...

Supplemental Figure 5
